# Role of long non-coding RNA in inflammatory bowel disease

**DOI:** 10.3389/fimmu.2024.1406538

**Published:** 2024-06-04

**Authors:** Yufei Hu, Yifan Lu, Yi Fang, Qizhe Zhang, Zhuoqun Zheng, Xiaojuan Zheng, Xiaohua Ye, Yanping Chen, Jin Ding, Jianfeng Yang

**Affiliations:** ^1^ Department of Gastroenterology, Affiliated Jinhua Hospital, Zhejiang University School of Medicine, Jinhua, Zhejiang, China; ^2^ Department of Geriatrics, Affiliated Jinhua Hospital, Zhejiang University School of Medicine, Jinhua, Zhejiang, China; ^3^ Department of Gastroenterology, Affiliated Hangzhou First People’s Hospital, School of Medicine, Westlake University, Hangzhou, Zhejiang, China

**Keywords:** inflammatory bowel disease, long non-coding RNA, intestinal barrier, immune homeostasis, biomarkers

## Abstract

Inflammatory bowel disease (IBD) is a group of recurrent chronic inflammatory diseases, including Crohn’s disease (CD) and ulcerative colitis (UC). Although IBD has been extensively studied for decades, its cause and pathogenesis remain unclear. Existing research suggests that IBD may be the result of an interaction between genetic factors, environmental factors and the gut microbiome. IBD is closely related to non-coding RNAs (ncRNAs). NcRNAs are composed of microRNA(miRNA), long non-coding RNA(lnc RNA) and circular RNA(circ RNA). Compared with miRNA, the role of lnc RNA in IBD has been little studied. Lnc RNA is an RNA molecule that regulates gene expression and regulates a variety of molecular pathways involved in the pathbiology of IBD. Targeting IBD-associated lnc RNAs may promote personalized treatment of IBD and have therapeutic value for IBD patients. Therefore, this review summarized the effects of lnc RNA on the intestinal epithelial barrier, inflammatory response and immune homeostasis in IBD, and summarized the potential of lnc RNA as a biomarker of IBD and as a predictor of therapeutic response to IBD in the future.

## 1 Introduction

Inflammatory bowel disease (IBD) is a group of immune-mediated chronic, non-specific and recurrent inflammatory diseases, which can involve the whole digestive tract, mainly including Crohn’s disease (CD) and ulcerative colitis (UC) (1). CD is characterized by affecting all layers of the intestinal wall in any part of the gastrointestinal tract in a discontinuous manner most commonly at the end of the terminal ileum or perianal region. The main symptoms are abdominal pain, weight loss, and varying degrees of diarrhea, often accompanied by complications such as stenosis, abscess, and fistula. In contrast, UC is characterized by inflammation that begins in the rectum and spreads proximally in a continuous manner, but the inflammation is limited to the mucosa and submucosa of the gut. Diarrhea, mucopurulent bloody stool and tenesmus are typical symptoms of active UC. As the incidence of IBD continues to increase globally, this disease is receiving increasing attention (2). Currently, the commonly used drugs for the treatment of IBD include aminosalicylic acids, glucocorticoids, immunosuppressants and biological inhibitors, which are mainly aimed at inducing and maintaining remission. 5-aminosalicylic acid (5-ASA), an active ingredient of aminosalicylic acid drugs, can treat IBD by anti-inflammatory and antioxidant effects (3). In UC patients, 5-ASA can also prevent colon cancer by regulating immunity and correcting intestinal flora imbalance. The adverse reactions of 5-ASA containing sulphapyridine (SP) were great, so the new preparation mesalazine was developed for clinical use. Mesalazine has a good effect on mild to moderate UC, but the effect on CD is not uncertain. The ECCO guidelines published in 2020 clearly state that it is not recommended for the induction and maintenance of remission in CD (4). Glucocorticoids can inhibit the activation of pro-inflammatory genes by regulating the transcription of anti-inflammatory protein genes and induce the degradation of pro-inflammatory gene mRNA to achieve anti-inflammatory effect (5). In the treatment of IBD with steroids, we should not only pay attention to hormone-related adverse reactions, which are related to the dose, administration method and duration of drugs (6), but also pay attention to hormone resistance. Immunosuppressants reduce the body’s immune response by inhibiting lymphocyte proliferation and activation. Because immunosuppressive agents need to be treated for a period of time to reach a stable plasma concentration, they are not suitable for the treatment of IBD in the acute phase. Studies have found that long-term use of immunosuppressants can increase the incidence of infection (7), which is an important factor leading to death in IBD patients. Biological agents can bind to specific targets and improve intestinal mucosal injury in IBD patients by blocking downstream inflammatory response and lymphocyte migration, thereby controlling symptoms and disease progression. At present, many biological agents have been used in the clinical stage for different pathways and targets. Common adverse reactions include infections, gastrointestinal reactions, allergies, headache and even severe infections, opportunistic infections and malignant tumors. Although the above drugs are effective at present, they have many limitations, because the etiology and pathogenesis of IBD are unclear. A growing body of evidence suggests that IBD may be the result of an interaction between genetic factors, environmental factors and the gut microbiome. Therefore, understanding its pathogenesis will help to explore better treatments.

RNA can be divided into messenger RNAs (mRNAs) that have the ability to encode proteins and non-coding RNAs (ncRNAs) that do not have the ability to encode proteins. Studies have found that only a small part of the 3 billion base pairs of the human genome has the ability to encode proteins, and the remaining about 98% of RNA is ncRNAs (8), including microrna (miRNA), small nucleolar RNA (snoRNA), long non-coding RNA (lncRNA), and circular RNA (circ RNA). NcRNAs play a key role in the regulation of some intracellular processes in prokaryotic and eukaryotic organisms. It involves the transcriptional and post-transcriptional levels of gene expression regulation, including mediating chromatin modification, regulating the activity of transcription factors, affecting the stability and processing and translation of mRNA, and regulating the function and localization of proteins. MiRNA, about 18–24 nucleotides in length, is post-transcriptional regulator that regulate post-transcriptional gene silencing to block translation by targeting the 3’ -untranslated region (3’UTR) of specific mRNA (9). At present, we have conducted the most thorough research on miRNA, and found differences in miRNA expression in IBD. In addition, miRNA plays a pro-inflammatory or anti-inflammatory role in regulating the pathogenesis of IBD, such as dysautophagy, activation of necrosis factor-ĸ B (NF-ĸB), and increased permeability of intestinal epithelium (10).

In contrast to miRNA, the role of lnc RNA in IBD has been poorly studied. Lnc RNAs are more than 200 nucleotide RNA molecules that are structurally similar to miRNA and may have A cap-like structure and polyA tails. According to current findings, they exhibit different functional roles, including regulating protein coding through chromatin remodeling, regulating gene expression through transcription, post-transcription, or guiding chromatin modification complexes to bind to specific genomic sites (11), and regulating protein activity and stability. The role of lnc RNA in immune dysfunction and autoimmune diseases such as rheumatoid arthritis (12), osteoarthritis (13), asthma (14), and type 1 diabetes mellitus (15) has attracted more and more attention. Existing studies have shown that lnc RNA plays an important role in the pathophysiology of IBD (16). Qiao et al. found the first association between lnc RNA and IBD in 2013 (17). They found that patients with clinically active CD had significantly higher levels of lnc RNA DQ786243 expression in peripheral blood cells compared with healthy controls or with inactive disease (17). Another lncRNA associated with CD pathophysiology is NRON, which is a non-coding repressor of NFAT. The molecule is involved in the RNA-protein complex, which prevents nuclear translocation of NFAT to inhibit NFAT. Leucine-rich repeat kinase-2 (LLRK2), a putative CD susceptibility gene, is also part of an RNA-protein complex. Some researchers have proposed a molecular mechanism of CD severity by finding that LRRK2-deficient mice are more sensitive to DSS-induced colitis (18). Similarly, another lncRNA, BC012900, was found to be significantly up-regulated in active UC tissues and stimulated by cytokines and pathogens through known IBD molecular pathways such as Toll-like and NOD2 receptors (19).

In this review, we highlight the role of lnc RNA in regulating gene expression and influencing the pathogenesis of IBD, as well as their potential as biomarkers and predictors of therapeutic response in IBD. By summarizing the effects of lnc RNA on intestinal epithelial barrier, inflammatory response and immune homeostasis in inflammatory bowel disease, this paper emphasizes the importance of lnc RNA in individualized therapy and treatment of IBD patients.

## 2 Roles of lnc RNAs in IBD

### 2.1 LncRNAs and intestinal barrier dysregulation

Intestinal barrier is mainly composed of intestinal epithelial cells and tight junction proteins between epithelial cells (20), which can block various harmful substances, such as intestinal microbiota, microbial products and antigens. Under normal circumstances, intestinal epithelial cells are constantly renewed to ensure the integrity of the intestinal epithelium. However, when hemorrhagic shock, acute pancreatitis, severe trauma and other conditions occur, the intestinal mucosa will appear ischemia or hypoxia, and the intestinal barrier will be damaged, causing the displacement of harmful substances and bacteria in the intestinal cavity, and even causing systemic inflammatory response syndrome or multiple organ dysfunction (21). Studies in patients with IBD have shown that the intestinal barrier function is disrupted regardless of whether the IBD disease is active or dormant. In addition, a decrease in compact connectin, increased apoptosis of epithelial cells, increased intestinal permeability, and disruption of intestinal barrier function were observed in patients with CD (22). Increased permeability of the intestinal epithelium was also observed during the inactive phase of the disease, suggesting a high probability of disease recurrence. In terms of intestinal barrier function, lnc RNAs can maintain intestinal homeostasis through various aspects, such as regulating intestinal epithelial regeneration and intestinal immunity (23, 24). At present, many studies have revealed the relationship between lnc RNAs and intestinal barrier in IBD, and further indicated the role of lncRNAs in IBD.

#### 2.1.1 Lnc RNA H19

**Figure 1:**
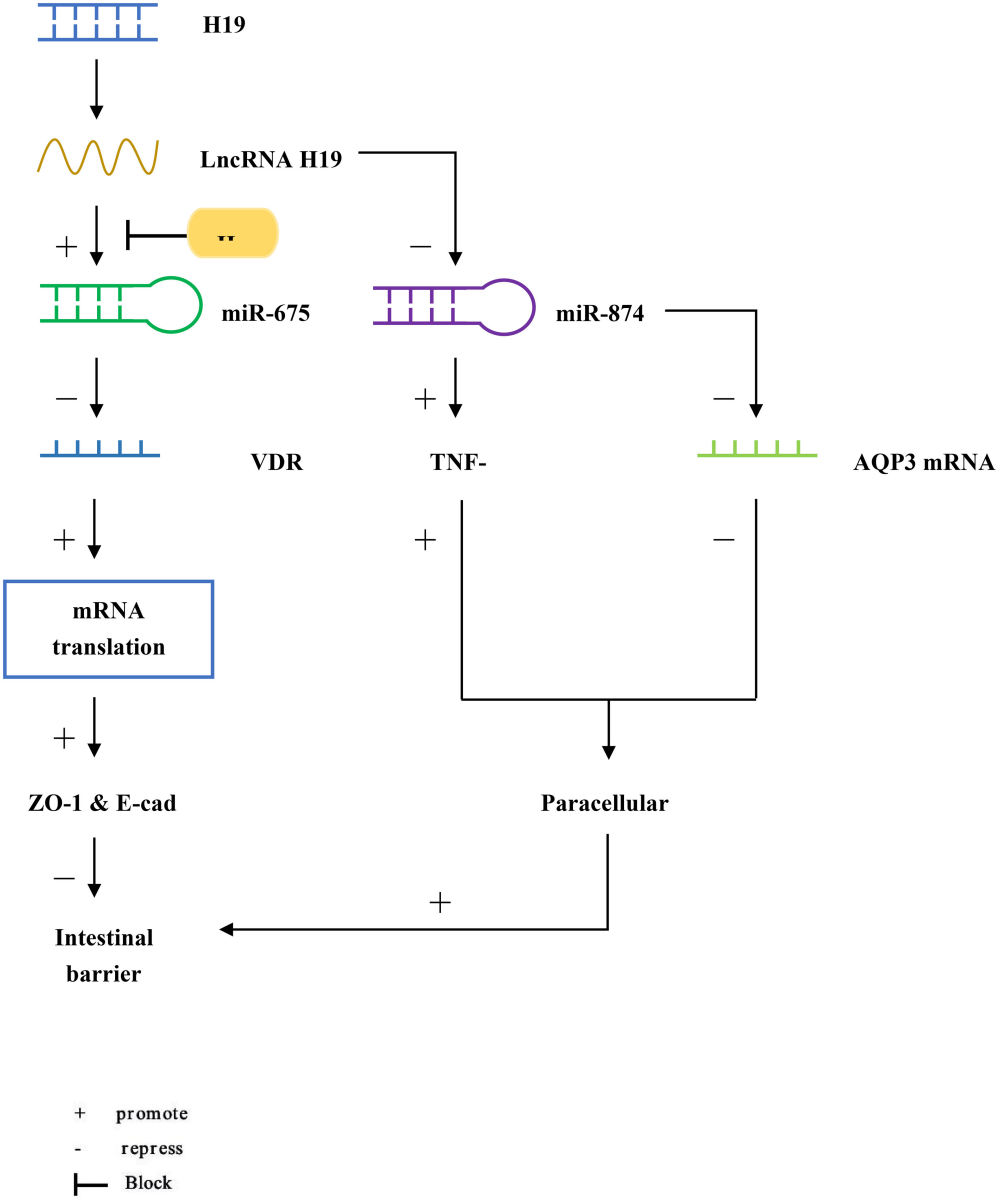
LncRNA H19 increases the abundance of miR-675, but it is blocked by HuR. miR-675 targets the 3’UTR of VDR mRNA to reduce the translation of ZO-1 and E-cad, causing intestinal barrier dysfunction. In addition, miR-874 targets the 3’UTR of AQP3 and increases the level of TNF-α, which enhances intestinal paracellular permeability and also causes intestinal barrier dysfunction.

H19 is an imprinted gene located on human chromosome 11p15.5 (**Figure 1**) (25). Lnc RNA H19 is mainly expressed in the embryo, generally decreased at birth, and significantly increased in tumors (26). We found that H19 promotes epithelial-mesenchymal transformation, and its knockdown can inhibit the growth of multiple myeloma cells through the NF-κB pathway, suggesting that H19 may play a role in the development of inflammatory diseases (27). Zou et al. reported that overexpression of Lnc RNA H19 increased the abundance of miR-675p and decreased the expression of zonula occludin 1 (ZO-1) and E-cadherin (E-cad), thus destroying the integrity of the intestinal barrier (28). This process is blocked by ubiquitous RNA-binding protein HuR. In another study, miR-675p was identified as a regulator targeting the 3’-untranslated region (3’UTR) of VDR mRNA in ulcerative colitis patient tissues (29). H19 expression exhibited a negative correlation with vitamin D receptor (VDR) mRNA expression (29). In the above studies, we can speculate that Lnc RNA H19 increases the abundance of miR-675, which targets VDR mRNA, causes the decrease of ZO-1 and E-cad expression, and finally destroys the intestinal barrier. Based on the function of H19 as a competing endogenous RNA (ceRNA), another target of action for Lnc RNA H19 in the intestinal barrier was discovered (30, 31). Zhi et al. found that during acute intestinal ischemia, miR-874 promoted paracellular permeability by changing the level of TNF-α and TJ proteins through targeting the 3’UTR of aquaporin 3 (AQP3), causing disruption of intestinal barrier function (32). Su et al. verified that Lnc RNA H19, as a ceRNA, regulated the expression of AQP3 by competing with miR-874, causing intestinal barrier dysfunction (33).

#### 2.1.2 PLnc RNA1

**Figure 2:**
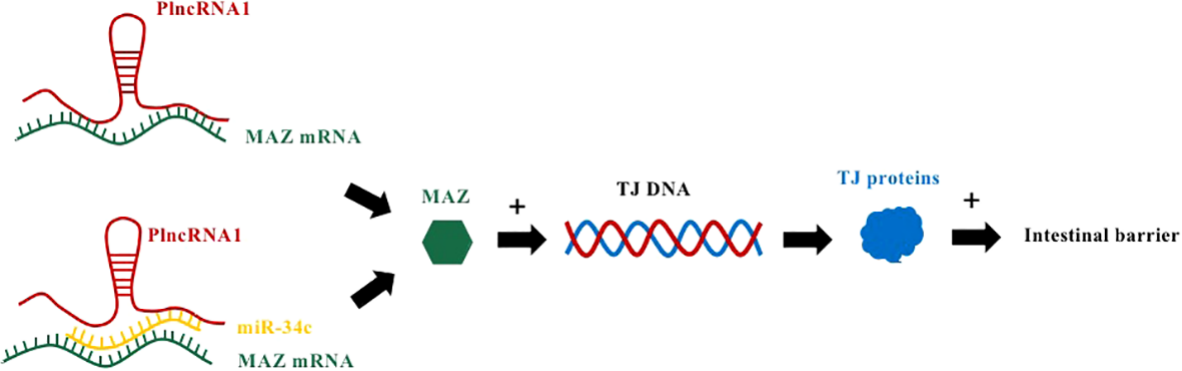
PlncRNA1 and miR-34c mediate IBD by affecting MAZ mRNA to regulate TJ proteins. PlncRNA1 directly interacts with MAZ mRNA and promotes the expression of MAZ. In addition, PlncRNA1 reduction affects the level of miR34c expressed by MAZ. MAZ interacts with the DNA sequences of the promoters of ZO-1, occludin, and claudin-5 to increase their expression.

Prostate cancer-upregulated long noncoding RNA1 (PLnc RNA1), a newly discovered lncRNA transcript, also known as CBR3 antisense RNA 1 (CBR3-AS1), located on chromosome 21q22.12, is upregulated in hepatocellular carcinoma (34), esophageal squamous cell carcinoma (35) and prostate cancer (**Figure 2**) (36). Overexpressed PLnc RNA1 has been reported to protect the intestinal epithelial barrier from damage by dextran sulfate sodium (DSS) (37). This is due to the fact that miR-34c binds to PLnc RNA1 and can directly target the 3’UTR of Myc-associated zinc-finger protein (MAZ), thereby regulating the expression of ZO-1 and occludin to regulate intestinal barrier function. In addition, the knockdown of PLnc RNA1 can reduce the expression of MAZ, and its effect can be reversed by down-regulating miR-34c, which indicates that PLnc RNA1 can also directly regulate the expression of MAZ (37).

#### 2.1.3 Lnc RNA SPRY4-IT1

**Figure 3:**
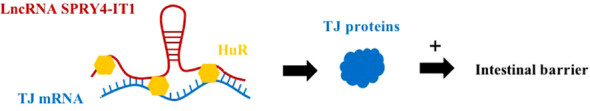
LncRNA SPRY4-IT1 has a protective effect on the intestinal barrier. SPRY4-IT1 can directly interact with TJ mRNA to promote the production of TJ proteins to protect the intestinal barrier. Alternatively, the SPRY4-IT1/HuR complex can also bind to TJ mRNA, which protects a protective effect on the intestinal barrier.

The SPROUTY4 intron transcript 1 (SPRY4-IT1) on chromosome 5q31.3 is transcribed from the SPRY4 gene (**Figure 3**) (38). Previous studies have shown that SPRY4-IT1 is essential for maintaining basal epithelial barrier function. Xiao et al. found in a study that lncRNA SPRY4-IT1 protected intestinal barrier function by stabilizing TJ mRNA and enhancing its translation (39). This study showed that by silencing SPRY4-IT1, the mRNA expression of TJ proteins ocgludin, claudin-1, claudin-3 and JAM-1 could be reduced, resulting in impaired intestinal barrier function (39). Conversely, increasing the level of SPRY4-IT1 increased the expression of TJ protein, which promoted the function of the intestinal barrier (39). SPRY4-IT1 directly interacts with TJ mRNA, and SPRY4-IT1 can also bind to RNA-binding protein HuR and then interact with TJ mRNA to increase the number of TJ protein and promote the function of intestinal barrier.

#### 2.1.4 Lnc RNA uc.173

**Figure 4:**
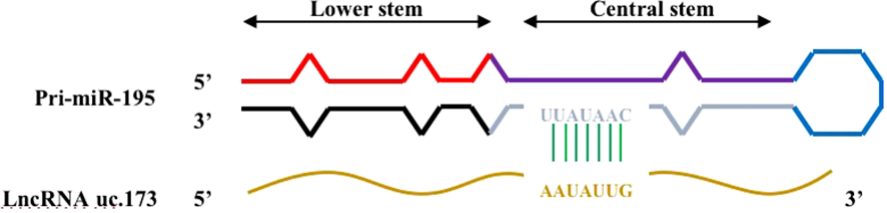
Complementary regions of pri-miR-195 interacting with lncRNA uc.173. There is a complementary region in the central stem region of pri-miR-195 that interacts with lncRNA uc.173.

Lnc RNA uc.173 is a transcribed ultra-conservative (T-UCR) region transcript representing the homologous region of human, rat, and mouse genomes (**Figure 4**) (40). A study determined the expression of 21 T-UCR genes, including uc.173, in mouse intestinal mucosa from the whole genome profile analysis, and found that increasing the expression of Lnc RNA uc.173 gene could increase the growth of intestinal epithelial cells, while decreasing the expression level reduced the renewal of intestinal epithelial cells (IECs) (41). This is due to the fact that lncRNA uc.173 destroys the pri-miR-195 transcript and induces degradation of miR-195 to down-regulate the expression of miRNA195, thereby stimulating intestinal epithelial cell renewal. It was later found that the complementary sequence capable of interacting with Lnc RNA uc.173 was located in the central stem region of pri-miR-195 (upstream of the 3’ terminal), indicating that this region can control the degradation of pri-miRNA. However, the relationship between miR-195 and IECs has not been clearly clarified so far.

#### 2.1.5 Lnc RNA BC012900

At present, the study of BC012900 is not thorough. A recent study reported that some cytokines such as TNF-α can regulate the expression of UC-related lncRNAs such as BC012900 (42). They further demonstrated that overexpression of BC012900 can significantly inhibit IEC cell proliferation and increase the sensitivity of IEC cells to apoptosis (42). However, the mechanism by which BC012900 further induces increased apoptosis in IEC cells is unclear. Since PPM1A expression is also increased in cells with increased BC012900 expression, they hypothesized that PPM1A expression may be one of the mechanisms by which BC012900 regulates apoptosis, based on previous reports that overexpression of PPM1A induces G2/M cell cycle arrest and apoptosis (43).

#### 2.1.6 Lnc RNA CRNDE

**Figure 5:**
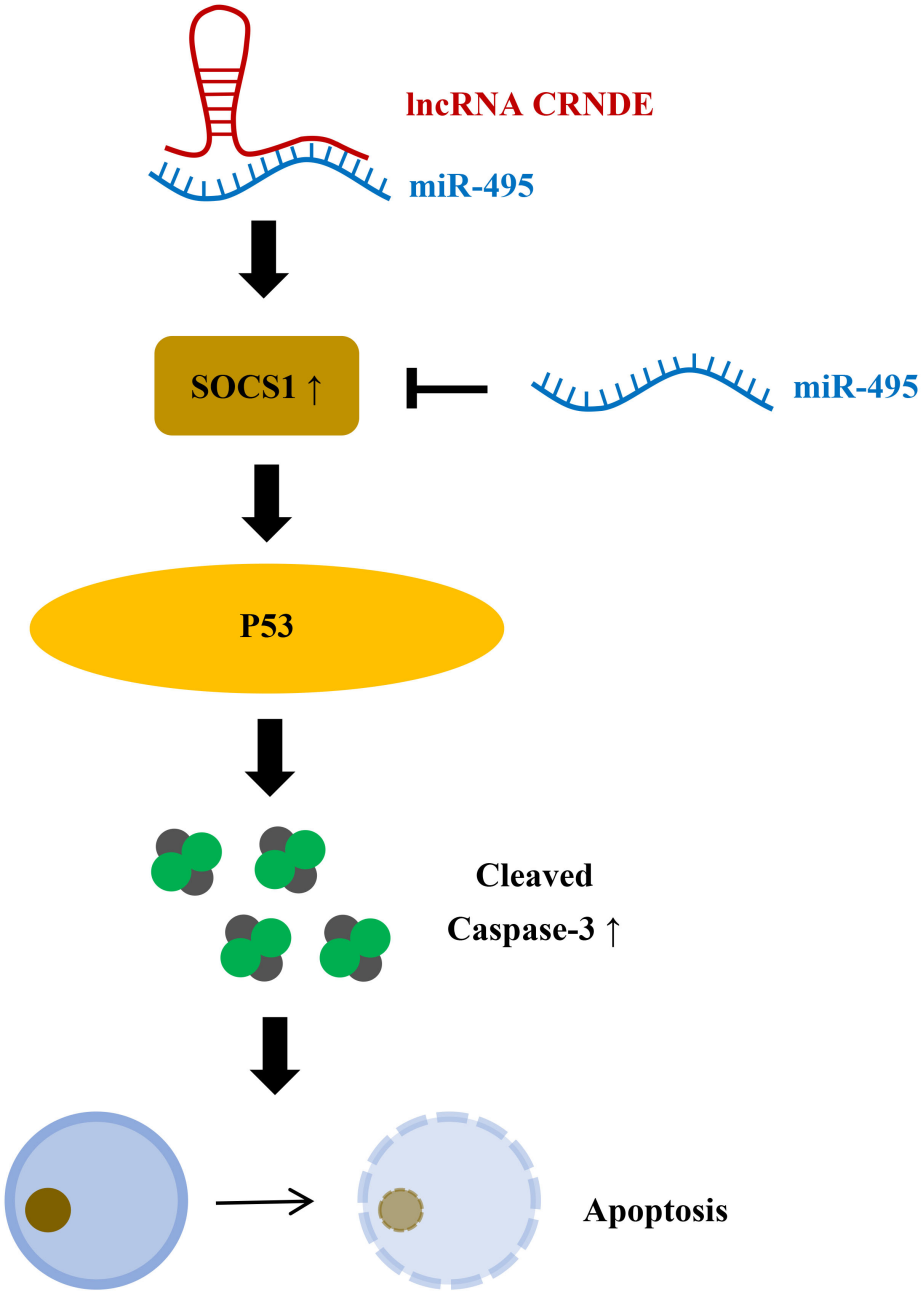
LncRNA CRNDE promotes apoptosis of colonic epithelial cells by inhibiting miR-495 and increasing SOCS1. miR-495 can inhibit SOCS1, and LncRNA CRNDE can increase SOCS1 by inhibiting miR-495 to increase P53 phosphorylation, increase activated Caspase-3, and promote apoptosis of colon epithelial cells.

CRNDE is a gene symbol for Colorectal Neoplasia differentientially expression (**Figure 5**) (44). It has been reported that Lnc CRNDE is highly expressed in colorectal cancer, hepatocellular carcinoma, and pancreatic cancer, and decreased in renal pheochromocytoma and ovarian cancer (45). CRNDE may affect tumorigenesis through regulation of miRNAs (46, 47). Studies have found that CRNDE may be related to the progression of IBD (48). In colitis tissue and colonic epithelial cell lines induced by dextran sulfate sodium(DSS), CRNDE can inhibit miRNA-495 expression and increase the expression of cytokine signaling pathway inhibitor (SOCS1) (48). By interfering with the expression of CRNDE, IBD symptoms were alleviated in mice. Chu et al. found that miRNA-495 expression decreased in UC, and miRNA-495 could prevent IEC apoptosis through JAK signaling (49). It has also been suggested that SOCS1 can induce IEC apoptosis by promoting IFN-γ (50). Suppressor of cytokine signaling (SOCS1), a member of the cytokine signaling pathway inhibitor family, is induced by interferon (IFN)-γ-mediated JAK signaling pathway. It is widely considered to be a protein that restricts cytokine receptor signaling (51). In animal models of colitis, p53 has been proven to mediate apoptosis of IECs (52). Cui et al. showed that SOCS1 increased p53 phosphorylation and promoted IFN-γ-induced apoptosis of IECs (50). Therefore, it can be speculated that lnc CRNDE regulates IEC apoptosis through the CRNDE/miR-495/SOCS1 axis, which also indicates that CRNDE is a potential target for the treatment of IBD.

#### 2.1.7 Lnc NEAT1

Lnc NEAT1 is a key component in building the ribo-riboprotein complex to regulate DNA-mediated activation of innate immune responses (53) and has also been found to play an important role in innate immune responses (54). Liu et al. found that NEAT1 was overexpressed in both DSS induced mouse models and tumor necrosis factor (TNF) -α-induced inflammatory cell models, and epithelial cell permeability increased in both mice and cell models compared to control cells (55). However, inhibition of NEAT1 can reverse its effects. This suggests that NEAT1 may affect the integrity and permeability of the intestinal epithelial barrier in patients with IBD. They also found that DSS could induce M1 macrophage activation, which was suppressed when NEAT1 expression was suppressed. Favre et al. demonstrated that low-dose photodynamic therapy (LDPDT) can improve T-cell-mediated colitis in mice (56). Subsequent studies have verified that PDT can alleviate DSS induced colitis in mice by regulating PI3K-AKT signaling pathway through Lnc NEAT1-miRNA204–5p axis, but whether PDT can alleviate clinical symptoms of IBD patients still needs further experimental verification (57).

#### 2.1.8 Other Lnc RNAs

CDKN2B-AS1 has more than 20 splicing variants, including typical splicing linear RNA and reverse splicing circular RNA molecules. Some studies have shown that downregulation of CDKN2B-AS1 can destroy Claudin-2 and cause the proliferation of intestinal epithelial cells, thus enhancing the intestinal barrier function (58). Another Lnc RNA, colon cancer-associated transcript 1 (CCAT1), was found to be overexpressed in IBD tissues. Ma et al. reported that CCAT1 expression was positively correlated with myosin light chain kinase (MLCK) (59). MLCK maintained the stability of MLCK mRNA by reducing the binding of miRNA-1853p to MLCK mRNA. MLCK and its phosphorylated products can increase intestinal permeability. CCAT1 and MLCK jointly accelerated the development of IBD (59).

The above studies suggest that lncRNAs not only regulate the apoptosis of epithelial cells in IBD, but also affect intestinal tight junction proteins and regulate the intestinal physical barrier through other mechanisms.

### 2.2 LncRNAs and immune homeostasis dysregulation

IBD is not only an inflammatory disease of intestinal mucosa, but also an abnormal immune disease caused by immune deficiency of intestinal mucosa (60). Nf-ĸB is an important immune response factor. When its inhibitory protein is phosphorylated and degraded by proteases, Nf-ĸB is transferred to the nucleus. It causes transcription of target genes such as interleukin-1β (IL-1β), interleukin-6 (IL-6), interleukin-8 (IL-8), and interferon γ (IFN-γ) (61). Studies have reported that colitis may be caused by excessive inflammatory events such as activation of NF-ĸB and increased expression of pro-inflammatory factors (62, 63). Since the gut contains complex immune cell populations and inflammatory networks, the precise etiology and pathogenesis of IBD have not been thoroughly studied (64). The intestinal immune system can maintain a complex balance between the intestinal proinflammatory and anti-inflammatory responses. Once the balance is broken, it may lead to the occurrence of IBD. Stimulated by interleukin-1b, IL-6, IL-8, and TNF, NF-ĸB triggers the transcription of pro-inflammatory cytokines (65). Currently, many studies have revealed that lnc RNAs can affect immune homeostasis in IBD.

#### 2.2.1 IFNG-AS1

IFNG-AS1 is a non-coding transcript located on human chromosome 12 adjacent to the IFNG gene (66). By comparing adult UC patients, Jurkat T cell model and mouse colitis model, Padua et al. found that IFNG-AS1 was associated with IBD susceptibility locus SNP rs7134599, and IFNG-AS1 could positively regulate the key inflammatory factor IFNG in CD4 T cells (66). Gomez et al. demonstrated that IFNG-AS1 regulates IFNG levels by binding to and actively regulating the histone methyltransferase complex MLL/SET1 (67). The MLL/SET1 complex can turn genes on and off at lysine 4 (K4) through methylation of histone H3 (68). Therefore, IFNG-AS1 may promote the action of Th1 cytokines (IFNG, IL2) and reduce the action of Th2 cytokines (IL10, IL13) through the MLL/SET1 complex (69). These results suggest that lnc RNA IFNG-AS1 is a potential target for the treatment of IBD patients.

#### 2.2.2 LINC01882

There is variation in the genetic locus of protein tyrosine phosphatase 2 (PTPN2) in IBD (70). PTPN2 regulates cytokine signaling by acting on a variety of phosphorylated proteins (71). Scharl et al. demonstrated that PTPN2 regulates autophagy in IECs and that there is a link between SNP rs2542151 and lower levels of PTPN2 protein in colon fibroblasts (72). SNPS at the PTPN2 locus were highly correlated with the DNA methylation level of four CpG sites downstream of PTPN2 and the expression level of the lncRNA LINC01882 downstream of these CpG sites (73). LINC01882, also known as LOC100996324 and RP11–973H3.4, is down-regulated in anti-CD3/CD28-activated CD4+ T cells and can inhibit T cell activation by inhibiting the expression of ZEB1, KLF12 and MAP2K4 to suppress IL-2 expression (73). It has been found that LINC01882 is involved in some autoimmune diseases including IBD. For example, LINC01882 ameliorates acute graft-versus-host disease (aGVHD) via skewing CD4+ T cell differentiation toward Treg cells (74). They also found that LINC01882 promoted Treg differentiation in CD4+ T cells via sponge let-7b-5p (74). This is a target for induction of immune tolerance, which offers the possibility of an effective therapeutic target for patients with aGVHD. LINC01882 is involved in IL-2 expression, which affects the immune response, homeostasis, and differentiation of a variety of lymphocytes, including Tregs. Changes in the number of Tregs contribute to the progression of autoimmune diseases. At present, there are few studies on LINC01882 and IBD, and their relationship still needs to be further explored.

#### 2.2.3 DQ786243

Tregs are important for maintaining intestinal self-tolerance, and their dysfunction is associated with CD and its degree of inflammation (65). Forkhead box P3 (Foxp3) is a major transcription factor controlling the development and function of Tregs. Kim et al. identified a T-cell receptor response enhancer in the first intron of Foxp3 that was dependent on a cyclic-AMP response element binding protein (CREB)/activating transcription factor (ATF) site overlapping a CpG island (75). Therefore, the generation, development, and function of Tregs are dependent on Foxp3 and CREB. Some studies have found that lncRNA DQ786243 and CREB are significantly overexpressed in patients with active CD compared with healthy people or patients with inactive CD (17). However, Foxp3 expression was decreased in inactive CD patients, and there was no significant difference between active CD and healthy people (17). DQ786243 may have a significant effect on the regulation of CREB and Foxp3 genes. Qiao et al. found that DQ786243 could promote CREB and Foxp3 expression and CREB phosphorylation after transfection in Jurkat cells (17). In addition, DQ786243, CREB and Foxp3 mRNAs were all associated with C-reactive protein (CRP), an important serum biomarker of inflammation (17). All these findings suggest that lncRNA DQ786243 is associated with CD, and DQ786243 may regulate the function of Tregs by affecting the expression levels of CREB and Foxp3.

#### 2.2.4 MEG3

Maternally expressed 3(MEG3), a currently concerned lncRNA, has been shown to have anti-inflammatory effects in a variety of inflammatory diseases (76, 77). Wang et al. found that lncRNA-MEG3 was expressed at low levels in a H2O2 -induced Caco-2 cell model and TNBS-induced ulcerative colitis in young rats (78). They injected the lncRNA MEG3 overexpression vector into the UC rat model and found that inflammatory cytokine levels and ROS release were significantly decreased, and IL-10 expression was significantly increased (78). IL-10 is a single-chain glycoprotein that can be produced by adaptive and innate immune cells. It has anti-inflammatory and immunomodulatory effects and can regulate the role of other cytokines in immune and inflammatory diseases. They also found that lncRNA MEG3 may inhibit the release of inflammatory cytokines and ROS by stimulating IL-10 expression (78). Their study similarly confirmed that pyroptosis and apoptosis can be triggered during the pathogenesis of UC, and lncRNA MEG3 can prevent both types of cell death (78). Some studies have reported that miR-98–5p can directly target IL-10 (79). Later they confirmed that lncRNA MEG3 positively regulates IL-10 expression. In addition, knockdown of miR-98–5p was previously shown to alleviate the symptoms of IBD (80). More importantly, the elevation of lncRNA MEG3 inhibited the upregulation of miR-985p expression and promoted the expression of IL-10 by sponging miR-98–5p in UC rats (78). In conclusion, lncRNA-MEG3 can alleviate UC by up-regulating miR-98–5p-loaded IL-10 expression, providing a new potential therapeutic strategy for UC treatment.

#### 2.2.5 LUCAT1

**Figure 6:**
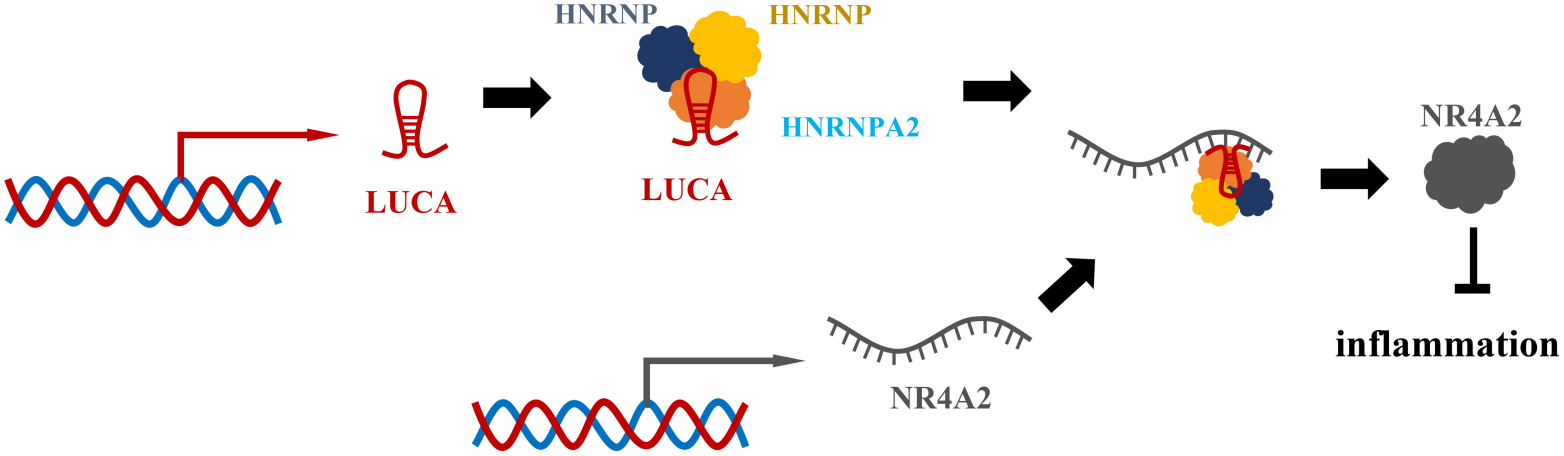
LUCAT1 regulates the splicing and stability of NR4A2 to suppress interferon and inflammatory factors. LUCAT1 binds to HNRNPA2B1, HNRNPC, HNRNPM and other proteins, participates in the splicing of NR4A2 mRNA, regulates the splicing and stability of NR4A2, and inhibits interferon and inflammatory factors.

LUCAT1 has previously been identified as a negative feedback regulator of type I interferon (IFN) and inflammatory cytokine expression in human bone marrow cells (**Figure 6**) (81). It was originally found in lung epithelial cells. Vierbuchen et al. identified the protein important in mRNA processing and alternative splicing as the LUCAT1 binding protein (82). These binding proteins include heterogeneous nuclear ribonucleoprotein (HNRNP) C, M and A2B1, which participate in mRNA splicing and processing, including an mRNA of anti-inflammatory gene NR4A2. At the same time, they found that cells lacking LUCAT1 altered splicing of selected immune genes. For example, splicing of nuclear receptor 4A2 (NR4A2) was particularly affected by lipopolysaccharide (LPS) stimulation (82). In cells lacking LUCAT1, the expression of NR4A2 is reduced and delayed, and the expression of immune genes is elevated in NR4A2-deficient cells. These observations suggest that LUCAT1 is induced to control the splicing and stability of NR4A2, which is partly responsible for the anti-inflammatory effects of LUCAT1. They also found that LUCAT1 levels were elevated in patients with chronic obstructive pulmonary disease (COPD) or IBD and that LUCAT1 levels correlated with disease severity (82). Another study revealed lnc RNA-mediated downregulation of innate immunity and inflammatory responses in the SARS-CoV-2 vaccination breakthrough infections (83). They found that LUCAT1 regulates NF-KB-dependent genes by regulating the JAK-STAT pathway in addition to IFN genes (83). In summary, LUCAT1 plays an important role in the regulation of inflammatory diseases. Therefore, LUCAT1 may be used as a potential biomarker and therapeutic target. It is necessary to conduct more studies on the role of LUCAT1 in inflammatory diseases, which can provide new treatment ideas for inflammatory diseases.

#### 2.2.6 Other Lnc RNAs

HIF1A-AS2 is the Roseburia intestinalis flagellin-induced lncRNA in gut epithelium. Quan et al. found that silencing HIF1A-AS2 abrogated the anti-inflammatory effect mediated by intestinal flagellin (84). They also found that HIF1A-AS2 inhibited inflammation by inactivating the NF-ĸB/JNK pathway and reducing the expression of cytokines such as TNF-a, IL-1b, IL-6, and IL-12 (84). Moreover, knockdown of HIF1A-AS2 significantly increased p65 and Jnk phosphorylation and fully abrogated flagellin-mediated anti-inflammatory effects *in vivo*. Their study provides new insights into the mechanisms by which lncrnas regulate flagellin-mediated resolution of colonic inflammation. Upstream stimulatory factor 1 (USF1) is a class of transcription factors related to coronary artery disease (CAD) (85), which can be used as a mediator to participate in the anti-inflammatory strategy for the treatment of acute lung injury (86). Activating transcription factor 2 (ATF2), a member of basic leucine zipper proteins, is widely expressed in various tissues and participates in inflammatory responses (87). Li et al. demonstrated the proinflammatory role of lncRNA HIF1A-AS2 in atherosclerosis (88). Down-regulation of lncRNA HIF1A-AS2 inhibits atherosclerotic inflammation by reducing the binding of USF1 to the promoter region of ATF2, thereby reducing the expression of ATF2 (88). Therefore, HIF1A-AS2 may be a negative regulator of intestinal inflammation and may be a new target for the treatment of IBD in the future.

LncRNA ANRIL, located on chromosome 9p21, shows significant down-regulation in IBD disease (89). Qiao et al. explored the role of ANRIL and its possible mechanistic studies after stimulating UC to cause inflammatory injury by lipopolysaccharide (LPS) treated human embryonic cells (FHCs) (90). They found that inhibition of ANRIL could negatively regulate miR-323b-5p to alleviate LPS-induced FHCs damage (90). In addition, their results also indicated that miR-323b-5p negatively regulated TLR4 expression (90). TLR4 was the target of miR-323b-5p. It has been shown to be upregulated in UC, causing inflammation and promoting UC development (91). Knockdown of TLR4 reversed the effect of miR-323b-5p in inhibiting LPS-induced injury in FHCs (90). TRL4 is the only TLR capable of activating the MyD88-dependent signaling pathway (92). MyD88 is not only a key downstream signaling ligand of TLR4 receptor complex, but also an important adaptor protein of NF-kB signaling pathway (93). NF-kB pathway is a central mediator involved in immune and inflammatory responses (94), which can play a therapeutic role in UC. Therefore, ANRIL may affect the development of UC by regulating the miR-323b-5p/TLR4/MyD88/NF-κB pathway, which provides new ideas for the treatment of UC in the future. Li et al. found many lncrnas differentially expressed in CD mucosa, indicating that these lncRNAs were all involved in the immune response (95). For example, lncRNA ATG induces stress and apoptotic protease activation in intestinal epithelial cells in the inflammatory environment of CD (96). Caspase-3-mediated cleavage of ATG16L1 is increased, leading to abnormal autophagy in intestinal epithelial cells (96). When the activity of DDX5 in Th17 cells is increased, a large number of lncRNA Rmrp is decomposed, which would bind to RORγ-t, thus causing the latter to undergo nuclear transport, acting on the corresponding promoter, and promoting the development of Th17. Th17 maturation has a protective effect on CD (42, 97, 98). In addition, ENST0000487539_1.1 levels are increased in the serum of CD patients, and they can regulate Treg cell function by regulating Foxp3 levels. They also hypothesized that these lncRNAs might be involved in the regulation of intestinal mucosal function through the genetic network of lncRNA-miRNA/TFs-mRNAs (95).

## 3 Lnc RNA as an IBD biomarker and a predictor of treatment response

The common methods for the diagnosis and treatment of IBD include clinical manifestations, imaging methods, histopathological examination, and endoscopic evaluation. Because clinical features of IBD vary among individuals, approximately one quarter of patients have extraintestinal manifestations before diagnosis (99). Histopathology and endoscopy are currently the “gold standard” for diagnosing IBD (100, 101). Because both of these methods rely heavily on skilled clinicians, diagnosis of IBD is difficult. At present, more and more researchers are more inclined to use some biomarkers, such as C-reactive protein (CRP), lactoferrin, calcaretin and so on. However, most current biomarkers reflect systemic inflammation, are present in many diseases, and lack a certain sensitivity and specificity, which can lead to treatment delays and further disease progression. Therefore, it is urgent to find out the biomarkers with high specificity and sensitivity. LncRNAs have been shown to be valuable diagnostic markers for various diseases due to their easy availability, stability, availability by common molecular biology techniques such as qRT-PCR, rapid detection, and quantification (102, 103). A variety of lncRNAs have been shown to be associated with IBD. By monitoring the change of lnc RNA level, the therapeutic effect and prognosis of IBD disease can be evaluated. Many lncRNAs can be used as biomarkers to evaluate clinical evaluation in patients with IBD.

Wang et al. demonstrated that lncRNA KIF9-AS1 and LINC01272 expression was significantly up-regulated, while lncRNA DIO3OS expression was significantly decreased in tissue and plasma samples of IBD patients compared with healthy controls (104). They also used ROC curve analysis to determine the specificity and sensitivity of KIF9-AS1, LINC01272 and DIO3OS, and the results showed that the area under the ROC curve (AUCs) of these three lncRNAs in IBD patients and healthy controls were mostly greater than 0.76 (104). Therefore, lncRNA KIF9-AS1, LINC01272 and DIO3OS may be potential diagnostic biomarkers for IBD. Ge et al. compared the expression of lncRNA ANRIL in CD patients and control group, and the results showed that the area under the curve (AUC) of lncRNA ANRIL expression to distinguish CD patients and control group was 0.803 (95%CI: 0.733–0.874) (105). They also found that lncRNA ANRIL expression could distinguish the active and remission stages of CD (105). They also found that lncRNA ANRIL expression could distinguish the active and remission stages of CD (105). In addition, lncRNA ANRIL expression was negatively correlated with CD disease risk, disease activity, and proinflammatory cytokine levels (105). With the development of the disease, complications such as fistula and stenosis may occur in the late stage of CD. Lncrnas can be used as biomarkers even when complications occur. Visschedijk et al. demonstrated that lncRNA RP11–679B19.1 is associated with recurrent fibrostenosis CD, but its specific mechanism of action remains unclear (106).

Not only can Lnc RNA serve as a biomarker, but it has also been shown to be a predictor of therapeutic response in IBD. Ge et al. found that changes in lncRNA ANRIL expression were associated with infliximab treatment response (105). The expression of lncRNA ANRIL increased in patients who achieved treatment response with infliximab, while it remained stable in patients who failed to achieve treatment response (105). Therefore, the change of intestinal mucosal lncRNA ANRIL is related to the response to infliximab treatment in CD patients, and its up-regulation can be used as a marker of the response to infliximab treatment in CD patients (105). Haberman et al. performed intestinal biopsies from IBD children undergoing endoscopy and treatment and found that the expression of LINC01272 and HNF4A-AS1 was significantly associated with more severe intestinal mucosal injury (89). Calprotectin S100A8 is the most commonly used tissue inflammation biomarker in clinical practice. They also found that HNF4A-AS1 was negatively correlated with the calprotectant S100A8, while LINC01272 was significantly positively correlated with the calprotectant S100A8 (89). HNF4A-AS1 is specifically expressed in epithelial cells, and LINC01272 is specifically expressed in monocytes, neutrophils, and myeloid dendritic cells (DC) (89). Therefore, targeted lncRNA-directed therapy may become potential new tissue-specific targets for RNA-based interventions. Glucocorticoids (GCs) are effective drugs for inducing remission in patients with IBD in clinical practice, which have anti-inflammatory and immunosuppressive effects (107). The level of lncRNA growth inhibition specific 5 (GAS5) was higher in patients with poor GCs response than in those with good response. Therefore, GAS5 may be associated with GCs resistance (108, 109). Some studies have found that the expression of lncRNA GAS5 is different between GCs sensitive cells and GCs resistant cells, and GAS5 is up-regulated in GCs resistant cells and accumulates more in the cytoplasm (110). In conclusion, lncRNA GAS5 can be considered as a novel pharmacogenomic marker that contributes to the personalization of GCs therapy (**Table 1**).

**Table 1:** LncRNAs proposed for IBD biomarkers and therapeutic predictors.

LncRNAs	Disease	Source	Method	Change	Application	Reference
KIF9-AS1	UC&CD	Colonic tissues & blood samples	qPCR	Upgrade	Biomarker between IBD and HC	(102)
LINC01272	UC&CD	Colonic tissues & blood samples	qPCR	Upgrade		
DIO3OS	UC&CD	Colonic tissues & blood samples	qPCR	Downgrade		
ANRIL	CD	Colonic tissues	qPCR	Downgrade	Biomarker between CD and HC, assessed the response to infliximab	(103)
RP11-679B19.1	CD	Ileal tissues	Immunochip	Upgrade	Associated with recurrent fibrous stenosis CD	(104)
LINC01272	CD	Ileal tissues	RNAseq	Downgrade	Associated with more severe intestinal mucosal injury	(105)
HNF4A-AS1	CD	Ileal tissues	RNAseq	Upgrade		
GAS5	UC&CD	Peripheral blood	qPCR	Upgrade	Marker of glucocorticoid therapy in children	(107)

UC, ulcerative colitis; CD, Crohn’s Disease; HC, healthy control; qPCR, quantitative real-time PCR; RNAseq, RNA sequencing.

## 4 Discussion

IBD is a recurrent chronic inflammatory disease in the gastrointestinal tract. Due to its increasing incidence, it has become a global health problem. Although the current drugs used in the clinical treatment of IBD are effective, they have many limitations. Given the complexity of IBD, *in vivo* approaches to investigate its etiology are essential. Although mouse models of IBD have been gradually developed and refined, the basic understanding of transcriptome differences in these models is still at an early stage, so the exact pathogenesis of these models is not fully understood. Although the exploration of lncRNAs in existing studies is still in the early stage, it has been pointed out that lncRNAs are involved in the pathogenesis of IBD. In IBD, lncRNAs can affect intestinal tight junction proteins, such as lncRNA H19, PLnc RNA1, lnc RNA SPRY4-IT1, etc. LncRNAs can regulate the apoptosis of epithelial cells, such as BC012900, lncRNA CRNDE and so on. LncRNAs also regulate the gut physical barrier through other mechanisms, such as the lncRNA CCAT1. In addition, lncRNAs can affect the immune response in IBD, such as lnc NEAT1, IFNG-AS1, LINC01882 and so on. With the development of molecular biology technology, monitoring the changes of lnc RNAs level can be used to evaluate the efficacy and prognosis of IBD. LncRNA KIF9-AS1, LINC01272 and DIO3OS may be potential diagnostic biomarkers for IBD. LncRNA ANRIL expression is negatively correlated with CD disease risk and disease activity, and is also related to the response to biological agents. Therefore, lncRNAs play an important role in regulating intestinal barrier and immune homeostasis. LncRNAs can not only be used as biomarkers and predictors of treatment response in IBD, but also as targets for IBD. Due to the complexity of IBD pathogenesis, a single lncRNA may not be able to fully explain IBD. Therefore, based on the close relationship between lncRNAs and IBD, it is essential to clarify the mechanism of lncRNAs in IBD and explore more promising treatment methods.

## Author contributions

YH: Writing – original draft, Writing – review & editing. YL: Writing – review & editing. YF: Writing – review & editing. QZ: Writing – review & editing. ZZ: Writing – review & editing. XZ: Writing – review & editing. XY: Writing – review & editing. YC: Methodology, Resources, Writing – review & editing. JD: Methodology, Resources, Writing – review & editing. JY: Methodology, Resources, Writing – review & editing.

## References

[1] Chang JT. Pathophysiology of inflammatory bowel diseases. N Engl J Med. (2020) 383:2652–64. doi: 10.1056/NEJMra2002697 33382932

[2] Ng SC Shi HY Hamidi N Underwood FE Tang W Benchimol EI. Worldwide incidence and prevalence of inflammatory bowel disease in the 21st century: a systematic review of population-based studies. Lancet. (2017) 390:2769–78. doi: 10.1016/S0140-6736(17)32448-0 29050646

[3] Beiranvand M. A review of the biological and pharmacological activities of mesalazine or 5-aminosalicylic acid (5-ASA): an anti-ulcer and anti-oxidant drug. Inflammopharmacology. (2021) 29:1279–90. doi: 10.1007/s10787-021-00856-1 34410540

[4] Torres J Bonovas S Doherty G Kucharzik T Gisbert JP Raine T. ECCO guidelines on therapeutics in crohn’s disease: medical treatment. J Crohns Colitis. (2020) 14:4–22. doi: 10.1093/ecco-jcc/jjz180 31711158

[5] Kadmiel M Cidlowski JA. Glucocorticoid receptor signaling in health and disease. Trends Pharmacol Sci. (2013) 34:518–30. doi: 10.1016/j.tips.2013.07.003 PMC3951203 23953592

[6] Keenan GF. Management of complications of glucocorticoid therapy. Clin Chest Med. (1997) 18:507–20. doi: 10.1016/s0272-5231(05)70398-1 9329873

[7] Toruner M Loftus EV Jr Harmsen WS Zinsmeister AR Orenstein R Sandborn WJ. Risk factors for opportunistic infections in patients with inflammatory bowel disease. Gastroenterology. (2008) 134:929–36. doi: 10.1053/j.gastro.2008.01.012 18294633

[8] Chen WX Ren LH Shi RH. Implication of miRNAs for inflammatory bowel disease treatment: Systematic review. World J Gastrointest Pathophysiol. (2014) 5:63–70. doi: 10.4291/wjgp.v5.i2.63 24891977 PMC4025074

[9] Filipowicz W Bhattacharyya SN Sonenberg N. Mechanisms of post-transcriptional regulation by microRNAs: are the answers in sight? Nat Rev Genet. (2008) 9:102–14. doi: 10.1038/nrg2290 18197166

[10] Innocenti T Bigagli E Lynch EN Galli A Dragoni G. MiRNA-Based Therapies for the treatment of inflammatory bowel disease: What are we still missing? Inflamm Bowel Dis. (2023) 29:308–23. doi: 10.1093/ibd/izac122 35749310

[11] Ghosal S Das S Chakrabarti J. Long noncoding RNAs: new players in the molecular mechanism for maintenance and differentiation of pluripotent stem cells. Stem Cells Dev. (2013) 22:2240–53. doi: 10.1089/scd.2013.0014 PMC3730374 23528033

[12] Song J Kim D Han J Kim Y Lee M Jin EJ. PBMC and exosome-derived Hotair is a critical regulator and potent marker for rheumatoid arthritis. Clin Exp Med. (2015) 15:121–6. doi: 10.1007/s10238-013-0271-4 24722995

[13] Steck E Boeuf S Gabler J Werth N Schnatzer P Diederichs S. Regulation of H19 and its encoded microRNA-675 in osteoarthritis and under anabolic and catabolic in *vitro* conditions. J Mol Med (Berl). (2012) 90:1185–95. doi: 10.1007/s00109-012-0895-y 22527881

[14] Tsitsiou E Williams AE Moschos SA Patel K Rossios C Jiang X. Transcriptome analysis shows activation of circulating CD8+ T cells in patients with severe asthma. J Allergy Clin Immunol. (2012) 129:95–103. doi: 10.1016/j.jaci.2011.08.011 21917308

[15] Mirza AH Kaur S Pociot F. Long non-coding RNAs as novel players in β cell function and type 1 diabetes. Hum Genomics. (2017) 11:17. doi: 10.1186/s40246-017-0113-7 28738846 PMC5525349

[16] Soroosh A Koutsioumpa M Pothoulakis C Iliopoulos D. Functional role and therapeutic targeting of microRNAs in inflammatory bowel disease. Am J Physiol Gastrointest Liver Physiol. (2018) 314:G256–62. doi: 10.1152/ajpgi.00268.2017 PMC5866423 29146677

[17] Qiao YQ Huang ML Xu AT Zhao D Ran ZH Shen J. LncRNA DQ786243 affects Treg related CREB and Foxp3 expression in Crohn’s disease. J BioMed Sci. (2013) 20:87. doi: 10.1186/1423-0127-20-87 24289115 PMC4174896

[18] Liu Z Lee J Krummey S Lu W Cai H Lenardo MJ. The kinase LRRK2 is a regulator of the transcription factor NFAT that modulates the severity of inflammatory bowel disease. Nat Immunol. (2011) 12:1063–70. doi: 10.1038/ni.2113 PMC4140245 21983832

[19] Wu F Huang Y Dong F Kwon JH. Ulcerative colitis-associated long noncoding RNA, BC012900, regulates intestinal epithelial cell apoptosis. Inflamm Bowel Dis. (2016) 22:782–95. doi: 10.1097/MIB.0000000000000691 26937624

[20] Wang J Ghosh SS Ghosh S. Curcumin improves intestinal barrier function: modulation of intracellular signaling, and organization of tight junctions. Am J Physiol Cell Physiol. (2017) 312:C438–45. doi: 10.1152/ajpcell.00235.2016 PMC5407015 28249988

[21] Mittal R Coopersmith CM. Redefining the gut as the motor of critical illness. Trends Mol Med. (2014) 20:214–23. doi: 10.1016/j.molmed.2013.08.004 PMC3959633 24055446

[22] Zeissig S Bürgel N Günzel D Richter J Mankertz J Wahnschaffe U. Changes in expression and distribution of claudin 2, 5 and 8 lead to discontinuous tight junctions and barrier dysfunction in active Crohn’s disease. Gut. (2007) 56:61–72. doi: 10.1136/gut.2006.094375 16822808 PMC1856677

[23] Yadav VK Kumar A Tripathi PP Gupta J. Long noncoding RNAs in intestinal homeostasis, regeneration, and cancer. J Cell Physiol. (2021) 236:7801–13. doi: 10.1002/jcp.30393 33899236

[24] Chen S He R He B Xu L Zhang S. Potential roles of exosomal lncRNAs in the intestinal mucosal immune barrier. J Immunol Res. (2021) 2021:7183136. doi: 10.1155/2021/7183136 34485536 PMC8413039

[25] Gabory A Ripoche MA Yoshimizu T Dandolo L. The H19 gene: regulation and function of a non-coding RNA. Cytogenet Genome Res. (2006) 113:188–93. doi: 10.1159/000090831 16575179

[26] Raveh E Matouk IJ Gilon M Hochberg A. The H19 long non-coding RNA in cancer initiation, progression and metastasis-a proposed unifying theory. Mol Cancer. (2015) 14:184. doi: 10.1186/s12943-015-0458-2 26536864 PMC4632688

[27] Pan JX. LncRNA H19 promotes atherosclerosis by regulating MAPK and NF-kB signaling pathway. Eur Rev Med Pharmacol Sci. (2017) 21:322–8. 28165553

[28] Zou T Jaladanki SK Liu L Xiao L Chung HK Wang JY. H19 long noncoding RNA regulates intestinal epithelial barrier function via MicroRNA 675 by Interacting with RNA-Binding Protein HuR. Mol Cell Biol. (2016) 36:1332–41. doi: 10.1128/MCB.01030-15 PMC4836219 26884465

[29] Chen SW Wang PY Liu YC Sun L Zhu J Zuo S. Effect of long noncoding RNA H19 overexpression on intestinal barrier function and its potential role in the pathogenesis of ulcerative colitis. Inflamm Bowel Dis. (2016) 22:2582–92. doi: 10.1097/MIB.0000000000000932 27661667

[30] Zhou X Ye F Yin C Zhuang Y Yue G Zhang G. The interaction between MiR-141 and lncRNA-H19 in regulating cell proliferation and migration in gastric cancer. Cell Physiol Biochem. (2015) 36:1440–52. doi: 10.1159/000430309 26160158

[31] Wang SH Wu XC Zhang MD Weng MZ Zhou D Quan ZW. Long noncoding RNA H19 contributes to gallbladder cancer cell proliferation by modulated miR-194–5p targeting AKT2. Tumour Biol. (2016) 37:9721–30. doi: 10.1007/s13277-016-4852-1 26803515

[32] Zhi X Tao J Li Z Jiang B Feng J Yang L. MiR-874 promotes intestinal barrier dysfunction through targeting AQP3 following intestinal ischemic injury. FEBS Lett. (2014) 588:757–63. doi: 10.1016/j.febslet.2014.01.022 24462679

[33] Su Z Zhi X Zhang Q Yang L Xu H Xu Z. LncRNA H19 functions as a competing endogenous RNA to regulate AQP3 expression by sponging miR-874 in the intestinal barrier. FEBS Lett. (2016) 590:1354–64. doi: 10.1002/1873-3468.12171 27059301

[34] Dong L Ni J Hu W Yu C Li H. Upregulation of long non-coding RNA PlncRNA-1 promotes metastasis and induces epithelial-mesenchymal transition in hepatocellular carcinoma. Cell Physiol Biochem. (2016) 38:836–46. doi: 10.1159/000443038 26906068

[35] Wang CM Wu QQ Li SQ Chen FJ Tuo L Xie HW. Upregulation of the long non-coding RNA PlncRNA-1 promotes esophageal squamous carcinoma cell proliferation and correlates with advanced clinical stage. Dig Dis Sci. (2014) 59:591–7. doi: 10.1007/s10620-013-2956-7 24337686

[36] Fang Z Xu C Li Y Cai X Ren S Liu H. A feed-forward regulatory loop between androgen receptor and PlncRNA-1 promotes prostate cancer progression. Cancer Lett. (2016) 374:62–74. doi: 10.1016/j.canlet.2016.01.033 26808578

[37] Chen T Xue H Lin R Huang Z. MiR-34c and PlncRNA1 mediated the function of intestinal epithelial barrier by regulating tight junction proteins in inflammatory bowel disease. Biochem Biophys Res Commun. (2017) 486:6–13. doi: 10.1016/j.bbrc.2017.01.115 28153728

[38] Katoh Y Katoh M. FGF signaling inhibitor, SPRY4, is evolutionarily conserved target of WNT signaling pathway in progenitor cells. Int J Mol Med. (2006) 17:529–32. doi: 10.3892/ijmm.17.3.529 16465403

[39] Xiao L Rao JN Cao S Liu L Chung HK Zhang Y. Long noncoding RNA SPRY4-IT1 regulates intestinal epithelial barrier function by modulating the expression levels of tight junction proteins. Mol Biol Cell. (2016) 27:617–26. doi: 10.1091/mbc.E15-10-0703 PMC4750922 26680741

[40] Nan A Zhou X Chen L Liu M Zhang N Zhang L. A transcribed ultraconserved noncoding RNA, Uc.173, is a key molecule for the inhibition of lead-induced neuronal apoptosis. Oncotarget. (2016) 7:112–24. doi: 10.18632/oncotarget.6590 PMC4807986 26683706

[41] Xiao L Wu J Wang JY Chung HK Kalakonda S Rao JN. Long Noncoding RNA uc.173 promotes renewal of the intestinal mucosa by inducing degradation of MicroRNA 195. Gastroenterology. (2018) 154:599–611. doi: 10.1053/j.gastro.2017.10.009 29042220 PMC5811324

[42] Zacharopoulou E Gazouli M Tzouvala M Vezakis A Karamanolis G. The contribution of long non-coding RNAs in inflammatory bowel diseases. Dig Liver Dis. (2017) 49:1067–72. doi: 10.1016/j.dld.2017.08.003 28869157

[43] Ofek P Ben-Meir D Kariv-Inbal Z Oren M Lavi S. Cell cycle regulation and p53 activation by protein phosphatase 2C alpha. J Biol Chem. (2003) 278:14299–305. doi: 10.1074/jbc.M211699200 12514180

[44] Graham LD Pedersen SK Brown GS Ho T Kassir Z Moynihan AT. Colorectal neoplasia differentially expressed (CRNDE), a novel gene with elevated expression in colorectal adenomas and adenocarcinomas. Genes Cancer. (2011) 2:829–40. doi: 10.1177/1947601911431081 PMC3278902 22393467

[45] Ellis BC Molloy PL Graham LD. CRNDE: A long non-coding RNA involved in cancer, neurobiology, and development. Front Genet. (2012) 3:270. doi: 10.3389/fgene.2012.00270 23226159 PMC3509318

[46] Chen Z Yu C Zhan L Pan Y Chen L Sun C. LncRNA CRNDE promotes hepatic carcinoma cell proliferation, migration and invasion by suppressing miR-384. Am J Cancer Res. (2016) 6:2299–309. PMC5088293 27822419

[47] Han P Li JW Zhang BM Lv JC Li YM Gu XY. The lncRNA CRNDE promotes colorectal cancer cell proliferation and chemoresistance via miR-181a-5p-mediated regulation of Wnt/β-catenin signaling. Mol Cancer. (2017) 16:9. doi: 10.1186/s12943-017-0583-1 28086904 PMC5237133

[48] Yang F Li XF Cheng LN Li XL. Long non-coding RNA CRNDE promotes cell apoptosis by suppressing miR-495 in inflammatory bowel disease. Exp Cell Res. (2019) 382:111484. doi: 10.1016/j.yexcr.2019.06.029 31251902

[49] Chu XQ Wang J Chen GX Zhang GQ Zhang DY Cai YY. Overexpression of microRNA-495 improves the intestinal mucosal barrier function by targeting STAT3 via inhibition of the JAK/STAT3 signaling pathway in a mouse model of ulcerative colitis. Pathol Res Pract. (2018) 214:151–62. doi: 10.1016/j.prp.2017.10.003 29129493

[50] Cui X Shan X Qian J Ji Q Wang L Wang X. The suppressor of cytokine signaling SOCS1 promotes apoptosis of intestinal epithelial cells via p53 signaling in Crohn’s disease. Exp Mol Pathol. (2016) 101:1–11. doi: 10.1016/j.yexmp.2016.05.011 27236107

[51] Calabrese V Mallette FA Deschênes-Simard X Ramanathan S Gagnon J Moores A. SOCS1 links cytokine signaling to p53 and senescence. Mol Cell. (2009) 36:754–67. doi: 10.1016/j.molcel.2009.09.044 20005840

[52] Dirisina R Katzman RB Goretsky T Managlia E Mittal N Williams DB. p53 and PUMA independently regulate apoptosis of intestinal epithelial cells in patients and mice with colitis. Gastroenterology. (2011) 141:1036–45. doi: 10.1053/j.gastro.2011.05.032 PMC3736614 21699775

[53] Imamura K Imamachi N Akizuki G Kumakura M Kawaguchi A Nagata K. Long noncoding RNA NEAT1-dependent SFPQ relocation from promoter region to paraspeckle mediates IL8 expression upon immune stimuli. Mol Cell. (2014) 53:393–406. doi: 10.1016/j.molcel.2014.01.009 24507715

[54] Morchikh M Cribier A Raffel R Amraoui S Cau J Severac D. HEXIM1 and NEAT1 long non-coding RNA form a multi-subunit complex that regulates DNA-mediated innate immune response. Mol Cell. (2017) 67:387–399.e385. doi: 10.1016/j.molcel.2017.06.020 28712728

[55] Liu R Tang A Wang X Chen X Zhao L Xiao Z. Inhibition of lncRNA NEAT1 suppresses the inflammatory response in IBD by modulating the intestinal epithelial barrier and by exosome-mediated polarization of macrophages. Int J Mol Med. (2018) 42:2903–13. doi: 10.3892/ijmm.2018.3829 30132508

[56] Favre L Borle F Velin D Bachmann D Bouzourene H Wagnieres G. Low dose endoluminal photodynamic therapy improves murine T cell-mediated colitis. Endoscopy. (2011) 43:604–16. doi: 10.1055/s-0030-1256382 21623559

[57] Wang K Zhang Z Liu K Yang X Zou H Zhou J. Neat1-miRNA204–5p-PI3K-AKT axis as a potential mechanism for photodynamic therapy treated colitis in mice. Photodiagnosis Photodyn Ther. (2018) 24:349–57. doi: 10.1016/j.pdpdt.2018.10.020 30385297

[58] Rankin CR Lokhandwala ZA Huang R Pekow J Pothoulakis C Padua D. Linear and circular CDKN2B-AS1 expression is associated with inflammatory bowel disease and participates in intestinal barrier formation. Life Sci. (2019) 231:116571. doi: 10.1016/j.lfs.2019.116571 31207308 PMC6897550

[59] Ma D Cao Y Wang Z He J Chen H Xiong H. CCAT1 lncRNA promotes inflammatory bowel disease Malignancy by destroying intestinal barrier via downregulating miR-185–3p. Inflamm Bowel Dis. (2019) 25:862–74. doi: 10.1093/ibd/izy381 30615124

[60] Geremia A Arancibia-Cárcamo CV. Innate lymphoid cells in intestinal inflammation. Front Immunol. (2017) 8:1296. doi: 10.3389/fimmu.2017.01296 29081776 PMC5645495

[61] Shih VF Tsui R Caldwell A Hoffmann A. A single NFκB system for both canonical and non-canonical signaling. Cell Res. (2011) 21:86–102. doi: 10.1038/cr.2010.161 21102550 PMC3193412

[62] Wang X Sun Y Zhao Y Ding Y Zhang X Kong L. Oroxyloside prevents dextran sulfate sodium-induced experimental colitis in mice by inhibiting NF-κB pathway through PPARγ activation. Biochem Pharmacol. (2016) 106:70–81. doi: 10.1016/j.bcp.2016.02.019 26947454

[63] Perše M Cerar A. Dextran sodium sulphate colitis mouse model: traps and tricks. J BioMed Biotechnol. (2012) 2012:718617. doi: 10.1155/2012/718617 22665990 PMC3361365

[64] Abraham C Cho JH. Inflammatory bowel disease. N Engl J Med. (2009) 361:2066–78. doi: 10.1056/NEJMra0804647 PMC3491806 19923578

[65] Boden EK Snapper SB. Regulatory T cells in inflammatory bowel disease. Curr Opin Gastroenterol. (2008) 24:733–41. doi: 10.1097/MOG.0b013e328311f26e 19125486

[66] Padua D Mahurkar-Joshi S Law IK Polytarchou C Vu JP Pisegna JR. A long noncoding RNA signature for ulcerative colitis identifies IFNG-AS1 as an enhancer of inflammation. Am J Physiol Gastrointest Liver Physiol. (2016) 311:G446–57. doi: 10.1152/ajpgi.00212.2016 PMC5076004 27492330

[67] Gomez JA Wapinski OL Yang YW Bureau JF Gopinath S Monack DM. The NeST long ncRNA controls microbial susceptibility and epigenetic activation of the interferon-γ locus. Cell. (2013) 152:743–54. doi: 10.1016/j.cell.2013.01.015 PMC3577098 23415224

[68] Popovic R Zeleznik-Le NJ. MLL: how complex does it get? J Cell Biochem. (2005) 95:234–42. doi: 10.1002/jcb.20430 15779005

[69] Rankin CR Shao L Elliott J Rowe L Patel A Videlock E. The IBD-associated long noncoding RNA IFNG-AS1 regulates the balance between inflammatory and anti-inflammatory cytokine production after T-cell stimulation. Am J Physiol Gastrointest Liver Physiol. (2020) 318:G34–40. doi: 10.1152/ajpgi.00232.2019 PMC6985849 31545920

[70] Franke A Balschun T Karlsen TH Hedderich J May S Lu T. Replication of signals from recent studies of Crohn’s disease identifies previously unknown disease loci for ulcerative colitis. Nat Genet. (2008) 40:713–5. doi: 10.1038/ng.148 18438405

[71] Doody KM Bourdeau A Tremblay ML. T-cell protein tyrosine phosphatase is a key regulator in immune cell signaling: lessons from the knockout mouse model and implications in human disease. Immunol Rev. (2009) 228:325–41. doi: 10.1111/j.1600-065X.2008.00743.x 19290937

[72] Scharl M Wojtal KA Becker HM Fischbeck A Frei P Arikkat J. Protein tyrosine phosphatase nonreceptor type 2 regulates autophagosome formation in human intestinal cells. Inflamm Bowel Dis. (2012) 18:1287–302. doi: 10.1002/ibd.21891 21987459

[73] Houtman M Shchetynsky K Chemin K Hensvold AH Ramsköld D Tandre K. T cells are influenced by a long non-coding RNA in the autoimmune associated PTPN2 locus. J Autoimmun. (2018) 90:28–38. doi: 10.1016/j.jaut.2018.01.003 29398253

[74] Teng Y Xia L Huang Z Yao L Wu Q. Long noncoding RNA LINC01882 ameliorates aGVHD via skewing CD4+ T cell differentiation toward Treg cells. Am J Physiol Cell Physiol. (2023) 324:C395–406. doi: 10.1152/ajpcell.00323.2022 36409171

[75] Kim HP Leonard WJ. CREB/ATF-dependent T cell receptor-induced FoxP3 gene expression: a role for DNA methylation. J Exp Med. (2007) 204:1543–51. doi: 10.1084/jem.20070109 PMC2118651 17591856

[76] Li Y Zhang S Zhang C Wang M. LncRNA MEG3 inhibits the inflammatory response of ankylosing spondylitis by targeting miR-146a. Mol Cell Biochem. (2020) 466:17–24. doi: 10.1007/s11010-019-03681-x 31894531

[77] Li G Liu Y Meng F Xia Z Wu X Fang Y. LncRNA MEG3 inhibits rheumatoid arthritis through miR-141 and inactivation of AKT/mTOR signalling pathway. J Cell Mol Med. (2019) 23:7116–20. doi: 10.1111/jcmm.14591 PMC6787440 31411001

[78] Wang Y Wang N Cui L Li Y Cao Z Wu X. Long non-coding RNA MEG3 alleviated ulcerative colitis through upregulating miR-98–5p-sponged IL-10. Inflammation. (2021) 44:1049–59. doi: 10.1007/s10753-020-01400-z 33394187

[79] Takuse Y Watanabe M Inoue N Ozaki R Ohtsu H Saeki M. Association of IL-10-regulating microRNAs in peripheral blood mononuclear cells with the pathogenesis of autoimmune thyroid disease. Immunol Invest. (2017) 46:590–602. doi: 10.1080/08820139.2017.1322975 28742402

[80] Peng Y Wang Q Yang W Yang Q Pei Y Zhang W. MiR-98–5p expression inhibits polarization of macrophages to an M2 phenotype by targeting Trib1 in inflammatory bowel disease. Acta Biochim Pol. (2020) 67:157–63. doi: 10.18388/abp.2020_5152 32242402

[81] Agarwal S Vierbuchen T Ghosh S Chan J Jiang Z Kandasamy RK. The long non-coding RNA LUCAT1 is a negative feedback regulator of interferon responses in humans. Nat Commun. (2020) 11:6348. doi: 10.1038/s41467-020-20165-5 33311506 PMC7733444

[82] Vierbuchen T Agarwal S Johnson JL Galia L Lei X Stein K. The lncRNA LUCAT1 is elevated in inflammatory disease and restrains inflammation by regulating the splicing and stability of NR4A2. Proc Natl Acad Sci USA. (2023) 120:e2213715120. doi: 10.1073/pnas.2213715120 36577072 PMC9910463

[83] Chattopadhyay P Mishra P Mehta P Soni J Gupta R Tarai B. Transcriptomic study reveals lncRNA-mediated downregulation of innate immune and inflammatory response in the SARS-CoV-2 vaccination breakthrough infections. Front Immunol. (2022) 13:1035111. doi: 10.3389/fimmu.2022.1035111 36466827 PMC9716354

[84] Quan Y Song K Zhang Y Zhu C Shen Z Wu S. Roseburia intestinalis-derived flagellin is a negative regulator of intestinal inflammation. Biochem Biophys Res Commun. (2018) 501:791–9. doi: 10.1016/j.bbrc.2018.05.075 29772233

[85] Laurila PP Soronen J Kooijman S Forsström S Boon MR Surakka I. USF1 deficiency activates brown adipose tissue and improves cardiometabolic health. Sci Transl Med. (2016) 8:323ra13. doi: 10.1126/scitranslmed.aad0015 26819196

[86] Tiruppathi C Soni D Wang DM Xue J Singh V Thippegowda PB. The transcription factor DREAM represses the deubiquitinase A20 and mediates inflammation. Nat Immunol. (2014) 15:239–47. doi: 10.1038/ni.2823 PMC4005385 24487321

[87] Li M Zhang D Ge X Zhu X Zhou Y Zhang Y. TRAF6-p38/JNK-ATF2 axis promotes microglial inflammatory activation. Exp Cell Res. (2019) 376:133–48. doi: 10.1016/j.yexcr.2019.02.005 30763583

[88] Li P Xing J Zhang J Jiang J Liu X Zhao D. Inhibition of long noncoding RNA HIF1A-AS2 confers protection against atherosclerosis via ATF2 downregulation. J Adv Res. (2020) 26:123–35. doi: 10.1016/j.jare.2020.07.015 PMC7584671 33133688

[89] Haberman Y BenShoshan M Di Segni A Dexheimer PJ Braun T Weiss B. Long ncRNA landscape in the ileum of treatment-naive early-onset crohn disease. Inflamm Bowel Dis. (2018) 24:346–60. doi: 10.1093/ibd/izx013 PMC6231367 29361088

[90] Qiao C Yang L Wan J Liu X Pang C You W. Long noncoding RNA ANRIL contributes to the development of ulcerative colitis by miR-323b-5p/TLR4/MyD88/NF-κB pathway. Biochem Biophys Res Commun. (2019) 508:217–24. doi: 10.1016/j.bbrc.2018.11.100 30477744

[91] Yu ZH Huang F Xu N Zhao DM Hu FA Liu J. Expression of Toll-like receptor 4, CD14, and NF-κB in Chinese patients with ulcerative colitis. J Immunoassay Immunochem. (2011) 32:47–56. doi: 10.1080/15321819.2010.538108 21253969

[92] Weighardt H Jusek G Mages J Lang R Hoebe K Beutler B. Identification of a TLR4- and TRIF-dependent activation program of dendritic cells. Eur J Immunol. (2004) 34:558–64. doi: 10.1002/eji.200324714 14768061

[93] Han LP Li CJ Sun B Xie Y Guan Y Ma ZJ. Protective effects of celastrol on diabetic liver injury via TLR4/MyD88/NF-κB signaling pathway in Type 2 diabetic rats. J Diabetes Res. (2016) 2016:2641248. doi: 10.1155/2016/2641248 27057550 PMC4745324

[94] Vallabhapurapu S Karin M. Regulation and function of NF-kappaB transcription factors in the immune system. Annu Rev Immunol. (2009) 27:693–733. doi: 10.1146/annurev.immunol.021908.132641 19302050

[95] Li N Shi R. Expression alteration of long non-coding RNAs and their target genes in the intestinal mucosa of patients with Crohn’s disease. Clin Chim Acta. (2019) 494:14–21. doi: 10.1016/j.cca.2019.02.031 30862513

[96] Murthy A Li Y Peng I Reichelt M Katakam AK Noubade R. A Crohn’s disease variant in Atg16l1 enhances its degradation by caspase 3. Nature. (2014) 506:456–62. doi: 10.1038/nature13044 24553140

[97] Calderón-Gómez E Bassolas-Molina H Mora-Buch R Dotti I Planell N Esteller M. Commensal-specific CD4(+) cells from patients with Crohn’s disease have a T-Helper 17 inflammatory profile. Gastroenterology. (2016) 151:489–500.e3. doi: 10.1053/j.gastro.2016.05.050 27267052

[98] Huang W Thomas B Flynn RA Gavzy SJ Wu L Kim SV. DDX5 and its associated lncRNA rmrp modulate TH17 cell effector functions. Nature. (2015) 528:517–22. doi: 10.1038/nature16193 PMC4762670 26675721

[99] Vavricka SR Rogler G Gantenbein C Spoerri M Prinz Vavricka M Navarini AA. Chronological order of appearance of extraintestinal manifestations relative to the time of IBD diagnosis in the swiss inflammatory bowel disease cohort. Inflamm Bowel Dis. (2015) 21:1794–800. doi: 10.1097/MIB.0000000000000429 26020601

[100] Sinh P Shen B. Endoscopic evaluation of surgically altered bowel in patients with inflammatory bowel diseases. Inflamm Bowel Dis. (2015) 21:1459–71. doi: 10.1097/MIB.0000000000000357 PMC4450893 25806847

[101] Vucelic B. Inflammatory bowel diseases: controversies in the use of diagnostic procedures. Dig Dis. (2009) 27:269–77. doi: 10.1159/000228560 19786751

[102] Bolha L Ravnik-Glavač M Glavač D. Long noncoding RNAs as biomarkers in cancer. Dis Markers. (2017) 2017:7243968. doi: 10.1155/2017/7243968 28634418 PMC5467329

[103] Ward M McEwan C Mills JD Janitz M. Conservation and tissue-specific transcription patterns of long noncoding RNAs. J Hum Transcr. (2015) 1:2–9. doi: 10.3109/23324015.2015.1077591 27335896 PMC4894084

[104] Wang S Hou Y Chen W Wang J Xie W Zhang X. KIF9-AS1, LINC01272 and DIO3OS lncRNAs as novel biomarkers for inflammatory bowel disease. Mol Med Rep. (2018) 17:2195–202. doi: 10.3892/mmr.2017.8118 PMC5783463 29207070

[105] Ge Q Dong Y Lin G Cao Y. Long noncoding RNA antisense noncoding RNA in the INK4 Locus correlates with risk, severity, inflammation and infliximab efficacy in Crohn’s disease. Am J Med Sci. (2019) 357:134–42. doi: 10.1016/j.amjms.2018.10.016 30665494

[106] Visschedijk MC Spekhorst LM Cheng SC van Loo ES Jansen BHD Blokzijl T. Genomic and expression analyses identify a disease-modifying variant for fibrostenotic Crohn’s disease. J Crohns Colitis. (2018) 12:582–8. doi: 10.1093/ecco-jcc/jjy001 29361163

[107] Ford AC Bernstein CN Khan KJ Abreu MT Marshall JK Talley NJ. Glucocorticosteroid therapy in inflammatory bowel disease: systematic review and meta-analysis. Am J Gastroenterol. (2011) 106:590–9. doi: 10.1038/ajg.2011.70 21407179

[108] Lucafò M Bravin V Tommasini A Martelossi S Rabach I Ventura A. Differential expression of GAS5 in rapamycin-induced reversion of glucocorticoid resistance. Clin Exp Pharmacol Physiol. (2016) 43:602–5. doi: 10.1111/1440-1681.12572 27001230

[109] Lucafo M De Iudicibus S Di Silvestre A Pelin M Candussio L Martelossi S. Long noncoding RNA GAS5: a novel marker involved in glucocorticoid response. Curr Mol Med. (2015) 15:94–9. doi: 10.2174/1566524015666150114122354 25601472

[110] Lucafò M Di Silvestre A Romano M Avian A Antonelli R Martelossi S. Role of the long non-coding RNA growth arrest-specific 5 in glucocorticoid response in children with inflammatory bowel disease. Basic Clin Pharmacol Toxicol. (2018) 122:87–93. doi: 10.1111/bcpt.12851 28722800

